# 超高效液相色谱-串联质谱法同时测定化妆品中100种糖皮质激素

**DOI:** 10.3724/SP.J.1123.2024.03011

**Published:** 2024-12-08

**Authors:** Dandan SHEN, Shanshan GONG, Bohai JIANG, Yuechen LI, Hengyue XUE, Bentao YU, Yifan KE, Zhiyuan LI, Xiqin MAO

**Affiliations:** 1.大连市药品检验检测院,辽宁 大连 116021; 1. Dalian Institute for Drug Control, Dalian 116021, China; 2.东疆海事局,天津 300461; 2. Dongjiang Maritime Safety Administration, Tianjin 300461, China; 3.上海爱博才思分析仪器贸易有限公司,北京 100015; 3. Shanghai AB Sciex Analytical Instrument Trading Co., Ltd., Beijing 100015, China

**Keywords:** 超高效液相色谱-串联质谱, 糖皮质激素, 化妆品, 非法添加, ultra performance liquid chromatography-tandem mass spectrometry (UPLC-MS/MS), glucocorticoid, cosmetic, illegally added

## Abstract

我国《化妆品安全技术规范》(2015年版)规定糖皮质激素为化妆品中禁用原料。本论文建立了超高效液相色谱-串联质谱(UPLC-MS/MS)同时检测化妆品中100种非法添加糖皮质激素的分析方法,涵盖了现行国家标准GB/T 24800.2-2009、GB/T 40145-2021和《化妆品安全技术规范》(2015年版)中列出的58种糖皮质激素,以及42种新型糖皮质激素,其中包含2种迄今未见文献报道的糖皮质激素美替诺龙醋酸酯和地塞米松9,11-环氧。样品用含0.2%(v/v)乙酸的饱和氯化钠溶液分散,用含0.2%(v/v)乙酸的乙腈提取,向乙腈提取液中加入等体积水析出非极性杂质,再加入10%(质量分数)亚铁氰化钾溶液和20%(质量分数)乙酸锌溶液(含0.4%(v/v)乙酸)作为沉淀剂进一步净化,选用Waters ACQUITY UPLC BEH C_18_色谱柱(150 mm×3.0 mm, 1.7 μm)进行色谱分离,以0.2%(v/v)乙酸水溶液-0.2%(v/v)乙酸甲醇溶液为流动相梯度洗脱。在电喷雾正离子模式下以动态多反应监测方式(MRM)测定,外标法定量。以MRM-信息依赖采集-增强子离子扫描模式(MRM-IDA-EPI)得到的增强子离子质谱图(EPI)进行阳性样品确证。结果显示:100种糖皮质激素在2.5~60 ng/mL范围内具有良好的线性关系,相关系数均大于0.99,除环索奈德在膏霜乳基质中出现基质抑制效应以外,其余99种糖皮质激素均无明显的基质效应。所有目标物的方法检出限和定量限分别为0.03 μg/g和0.1 μg/g。在3个不同的添加水平下(1倍、2倍和8倍定量限),水基质中目标化合物的回收率为79.6%~114.4%, RSD为0.7%~9.4%(*n*=6),膏霜乳基质中目标化合物的回收率为79.5%~112.1%, RSD为0.7%~12.9%(*n*=6)。筛查了47种实际样品,在3个阳性样品中检出了5种糖皮质激素,并用MRM-IDA-EPI进行了确证。本检测方法操作简便、高效,适用于化妆品中100种糖皮质激素的快速筛查和测定,可填补化妆品中糖皮质激素的监管空白,为化妆品的质量监管提供了有力的技术手段。

## Abstract (en)

Adding glucocorticoids to cosmetics is strictly prohibited under Safety and Technical Specification for Cosmetics (2015) regulations. Accordingly, a method for simultaneously determining 100 glucocorticoids in cosmetics using ultra performance liquid chromatography-tandem mass spectrometry (UPLC-MS/MS) was developed. Apart from the 58 glucocorticoids listed in the two current national standards (GB/T 24800.2-2009 and GB/T 40145-2021) and the Safety and Technical Specification for Cosmetics (2015) regulations, 42 new glucocorticoids, including unreported metenolone acetate and dexamethasone 9,11-epoxide, were included in this method. Samples were dispersed in saturated sodium chloride containing 0.2% (v/v) acetic acid, and extracted with acetonitrile containing 0.2%(v/v) acetic acid. After that, an equal volume of water was added to the acetonitrile extraction solution to precipitate non-polar impurities. The solution was subsequently further purified using 10%(mass fraction) potassium ferrocyanide solution and 20%(mass fraction) zinc acetate solution containing 0.4% (v/v) acetic acid as precipitants. The sample solution was finally loaded onto a Waters ACQUITY UPLC BEH C_18_ column (150 mm×3.0 mm, 1.7 μm) and gradient-eluted using aqueous and methanolic 0.2% (v/v) acetic acid solutions as mobile phases. The target compounds exhibited highly intense signals in positive electrospray-ionization mode. All compounds were analyzed in multiple reaction monitoring mode (MRM) and quantified using the external standard method. Rimexolone and hydrocortisone hemisuccinate hydrate exhibited peaks corresponding to their [M-H_2_O+H]^+^ base ions, whereas the other 98 glucocorticoids exhibited [M+H]^+^ peaks. All positive samples were confirmed by their automated MRM-based information dependent acquisition (IDA) enhanced product ions (EPIs). Highly sensitive secondary mass spectra were acquired when EPI mode was combined with IDA and when target compounds in positive samples were located in MRM mode, with compounds further confirmed by matching their secondary mass spectra with those of the corresponding standard glycocorticoids using the Library Search function in SCIEX OS software.

Matrix effects (MEs) were investigated in water- and cream-based cosmetics by adding standard compounds to the extracted-matrix solutions. Ciclesonide exhibited no obvious matrix effect with a ME of 3.4% in the water-based matrix whereas it exhibited a serious inhibitory effect with a ME of 30.6% in the cream-based matrix. The remaining 99 target compounds exhibited MEs of -7.9%-12.4%, which are not significant because they are below 15%, further confirming that the sample-pretreatment method is effective and feasible. Therefore, ciclesonide in a cream-based matrix was quantified using a matrix-matched standard curve, while the others were quantified using solvent standard curves. The 100 glucocorticoids exhibited good linearities with correlation coefficients (*r*) of >0.99 in the range of 2.5-60 ng/mL. The limits of detection (LODs) and limits of quantification (LOQs) of this method were 0.03 and 0.1 μg/g, respectively. Recovery tests were performed at three spiked levels, namely one-, two-, and eight-times the LOQs. The 100 compounds exhibited recoveries of 79.6%-114.4% with relative standard deviations (RSDs) of 0.7%-9.4% (*n*=6) in water-based cosmetics; in comparison, they exhibited recoveries of 79.5%-112.1% with RSDs of 0.7%-12.9% (*n*=6) in cream-based cosmetics. Three cream-based positive samples were identified among 47 batches of cosmetics samples (including 25 batches of cream and lotion-based cosmetics, and 22 batches of water-based cosmetics), with quantitative analysis revealing five positive compounds: triamcinolone acetonide (115.1 μg/g), triamcinolone acetonide 21-acetate (0.6 μg/g), clobetasol propionate (24.5 μg/g), dexamethasone (2.7 μg/g), and dexamethasone-17-acetate (210.3 μg/g). These five compounds were further confirmed using MRM-IDA-EPI. The secondary mass spectra of the five compounds showed high matching degrees (>96%) with those of the corresponding standard glucocorticoids in the library database.

In conclusion, the method developed in this study is simple, practical, inexpensive, highly efficient, and suitable for qualitatively and quantitatively screening and analyzing 100 glucocorticoids in cosmetics, thereby filling supervisory gaps and greatly improving the detectability of glucocorticoids illegally added to cosmetics.

糖皮质激素可降低皮肤毛细血管的通透性,减少渗出和细胞浸润,具有抗炎、抗过敏、免疫抑制、抗增生等作用,临床上应用非常广泛。使用非法添加糖皮质激素的化妆品短时间内可使皮肤出现光滑、白嫩的假象,美容效果显著,在利益驱使下化妆品中非法添加糖皮质激素的案例时有发生^[1,2]^。2021年1月至2024年2月,国家药品监督管理局官网上《国家药监局关于化妆品检出禁用原料的通告》^[3,4]^中涉及糖皮质激素的不合格化妆品共26批,其中2批为儿童化妆品,检出量为0.14~287 μg/g,不合格产品多宣称具有祛痘、修护、祛斑、抗皱的功效,包括水类、膏霜乳类、凝胶类、面膜类。据报道使用糖皮质激素4周以上会出现皮肤依赖反应,一旦停用就会缺水、起皮、毛细血管暴露,真菌感染率升高,进而引起继发性的皮肤炎症性疾病,治疗难度很大,对消费者健康造成严重伤害。

《化妆品安全技术规范》(2015年版)^[5]^将糖皮质激素列为化妆品禁用组分,并规定了50种糖皮质激素的检测方法,另有国家标准GB/T 24800.2-2009^[6]^和GB/T 40145-2021^[7]^分别规定了41种和11种糖皮质激素的检测方法,3个法定检验方法共涵盖糖皮质激素58种。现有法定标准检测方法均为靶向检测,靶向检测的缺点是无法检测靶标以外的物质。糖皮质激素是一类结构非常容易改造的化合物,经过结构改造的糖皮质激素处于监管盲区,很难被发现,在化妆品中检出法定标准检验范围之外的糖皮质激素的案例已有多篇文献报道^[8⇓-10]^,以此逃避监管的行为已经实际存在,说明糖皮质激素的法定标准检验已经存在严重风险,亟需开发更高通量糖皮质激素检测方法填补漏洞,作为打击糖皮质激素违法添加行为的有力武器。

目前糖皮质激素的检测方法多为液相色谱-串联质谱法(LC-MS/MS)^[9⇓⇓⇓⇓-14]^和液相色谱-高分辨质谱法(LC-HRMS)^[15⇓⇓⇓⇓-20]^,因高分辨质谱设备昂贵,LC-HRMS的普及率不如低分辨的LC-MS/MS,目前化妆品中糖皮质激素的法定标准检验方法均为LC-MS/MS和薄层色谱法。赵倩茹等^[10]^、马亮波等^[11]^、罗辉泰等^[12]^、刘红等^[13]^分别报道了83种、42种、81种和73种糖皮质激素的检测方法,目前已见方法报道的糖皮质激素种类已达90余种。化妆品样品前处理形式多样,包括溶剂萃取^[10]^、液液萃取^[6,14,19,21]^、固相萃取^[11,13,17]^、QuEChERs净化技术^[12,14,21]^、在线净化^[18]^等技术,色谱柱主要使用C_18_色谱柱^[5⇓-7,11,13,15⇓⇓⇓-19]^和PFP色谱柱^[10,12,14,20]^。

为了填补化妆品中糖皮质激素的监管空白,为化妆品中糖皮质激素非法添加提供更高效的检测方法和更有力的监管手段,本文通过优化化妆品样品前处理方法(双液相萃取、加沉淀剂、加水、加酸)、液相色谱条件(流动相、色谱柱、洗脱梯度)和质谱条件(母离子、子离子、碰撞能等质谱参数),建立了一种化妆品中100种糖皮质激素同时测定的方法,方法涵盖当前法定标准检验方法中的58种糖皮质激素,增加了42种新型糖皮质激素,包括2种未见于公开文献资料中的糖皮质激素:美替诺龙醋酸酯和地塞米松9,11-环氧。

## 1 实验部分

### 1.1 仪器、试剂与材料

AB Sciex QTRAP 5500+液相色谱-质谱联用仪(美国AB SCIEX公司); IKA MS3型多管旋涡混合仪(德国IKA公司); CF16RXII型高速离心机(日本HITACHI公司)。

100种糖皮质激素对照品,纯度均大于95%,分别购自中国食品药品检定研究院、美国药典委员会、加拿大TRC公司、德国Dr. Ehrenstorfer公司、中国CATO公司、中国阿拉丁公司、中国曼哈格公司、中国阿尔塔公司和中国北欣景溢公司。

甲醇(质谱级,瑞典OCEANPAK公司);乙腈(质谱级,美国Merck公司);乙酸(质谱级,美国Fisher公司);氯化钠、乙酸锌和亚铁氰化钾(优级纯,天津市光复科技发展有限公司);实验用水为屈臣氏蒸馏制法饮用水。

滤膜:孔径为0.22 μm的有机滤膜。

### 1.2 样品前处理

准确称取0.25 g样品(精确至0.001 g)于10 mL具塞刻度离心管中,先加入4 mL含0.2%(v/v)乙酸的饱和氯化钠溶液(经乙腈饱和后使用)涡旋混匀后,再加入5 mL含0.2%(v/v)乙酸的乙腈,充分涡旋1 min后于5000 r/min离心10 min。移取上层清液至10 mL离心管中,加入含0.2%(v/v)乙酸的水约4 mL,加入10%(质量分数)亚铁氰化钾溶液和20%(质量分数)乙酸锌溶液(含0.4%(v/v)乙酸)各0.25 mL,加入含0.2%(v/v)乙酸的水定容至10 mL,摇匀,充分涡旋1 min,于10000 r/min离心5 min,经滤膜过滤,弃去初滤液1 mL,取续滤液转入进样瓶中待测。

### 1.3 标准溶液的配制

储备液:分别准确称取100种糖皮质激素标准物质(精确至0.0001 g)10 mg至10 mL棕色容量瓶中,用甲醇定容至刻度,配制质量浓度为1.0 mg/mL的储备液,于-20 ℃冷冻保存。

混合标准中间液:分别准确移取糖皮质激素的标准储备液适量,用乙腈定容,配制质量浓度为1 μg/mL的混合标准中间液。

混合标准工作溶液:准确移取混合标准中间液25、100、200、400、600 μL至10 mL容量瓶中,用含0.2%(v/v)乙酸的50%(v/v)乙腈水溶液定容至刻度,配制质量浓度分别为2.5、10、20、40、60 ng/mL的混合标准工作溶液。

基质标准工作溶液的配制:准确称取与待测样基质相近的空白基质0.25 g,移取混合标准中间液25、100、200、400、600 μL至10 mL容量瓶中,按1.2节处理,得到质量浓度分别是2.5、10、20、40、60 ng/mL的基质标准工作溶液。

### 1.4 分析条件

色谱柱:Waters ACQUITY UPLC BEH C_18_色谱柱(150 mm×3.0 mm, 1.7 μm);流动相A: 0.2%(v/v)乙酸水溶液;流动相B: 0.2%(v/v)乙酸甲醇溶液;流速:0.4 mL/min;进样量:5 μL;柱温:40 ℃;梯度洗脱程序:0~2 min, 70%A~60%A; 2~8 min, 60%A~50%A; 8~11 min, 50%A; 11~20 min, 50%A~35%A; 20~26 min, 35%A~5%A; 26~31 min, 5%A; 31~31.1 min, 5%A~70%A; 31.1~35 min, 70%A。

离子源:电喷雾电离源(ESI源);扫描方式:正离子扫描;检测方式:多反应监测(MRM)模式;气帘气(CUR): 240 kPa;碰撞气(CAD): 60 kPa;离子源电压:5500 V;温度:550 ℃; Gas1(喷雾气): 380 kPa; Gas2(辅助加热气): 410 kPa。其他质谱参数见表1。

**表1:** 100种糖皮质激素的CAS号、保留时间及质谱参数

No.		Compound		CAS No.			Parent ion (*m/z*)			Product ions (*m/z*)			*t*_R_/ min		Declustering potential/V		Collision energies/V	
1	deprodone (迪普罗酮)				20423-99-8			327.2			147.1^*^/171.1			16.53		110		33/33
2	medrysone (甲羟松)				2668-66-8			345.2			327.3^*^/135.2			24.09		100		20/27
3	methenolone acetate (美替诺龙醋酸酯)				434-05-9			345.3			187.2^*^/303.4			26.77		125		30/25
4	cortexolone (脱氧可的松)				152-58-9			347.3			108.9^*^/97.0			18.19		126		45/47
5	rimexolone (瑞美松龙)				49697-38-3			353.2			121.1^*^/173.1			25.85		100		45/31
6	prednisone (泼尼松)				53-03-2			359.2			341.2^*^/147.2			11.35		80		15/35
7	cortisone (可的松)				53-06-5			361.2			163.2^*^/121.1			11.89		80		34/47
8	prednisolone (泼尼松龙)				50-24-8			361.2			343.2^*^/147.2			13.71		80		14/34
9	hydrocortisone (氢化可的松)				50-23-7			363.2			121.1^*^/105.0			13.68		80		31/68
10	meprednisone (甲基泼尼松)				1247-42-3			373.2			355.2^*^/147.1			16.53		70		17/31
11	dexamethasone 9,11-epoxide (地塞米松9,11-环氧)				24916-90-3			373.1			355.3^*^/337.3			17.79		90		17/16
12	methylprednisolone (甲基泼尼松龙)				83-43-2			375.2			357.1^*^/161.1			18.02		66		14/28
13	16*α*-hydroxyprednisolone (16*α*-羟基泼尼松龙)				13951-70-7			377.2			359.2^*^/147.1			9.41		70		17/30
14	6*α*-methyl hydrocortisone (6*α*-甲基氢化可的松)				1625-39-4			377.3			341.4^*^/323.3			18.12		130		24/25
15	fluorometholone (氟米龙)				426-13-1			377.2			279.3^*^/321.3			19.21		80		22/18
16	fluocortolone (氟可龙)				152-97-6			377.2			121.0^*^/147.0			19.93		70		41/32
17	desoximetasone (去羟米松)				382-67-2			377.2			171.0^*^/147.0			20.17		70		29/28
18	isoflupredone (异氟泼尼松)				338-95-4			379.3			359.2^*^/341.2			12.39		90		14/18
19	fluprednisolone (氟泼尼龙)				53-34-9			379.1			341.3^*^/323.2			12.68		95		16/17
20	fludrocortisone (氟氢可的松)				127-31-1			381.1			239.2^*^/181.2			12.98		150		35/40
21	deflazacort (21-去乙酰基地夫可特)				13649-88-2			384.2			126.1^*^/147.1			19.20		120		43/38
22	betamethasone base (倍他米松)				378-44-9			393.2			355.0^*^/146.8			17.25		56		18/37
23	dexamethasone (地塞米松)				50-02-2			393.2			355.0^*^/146.8			17.43		53		17/40
24	paramethasone (帕拉米松)				53-33-8			393.2			337.2^*^/171.1			17.76		80		16/34
25	cloprednol (氯泼尼醇)				5251-34-3			393.2			205.0^*^/271.1			20.11		80		32/25
26	triamcinolone (曲安西龙)				124-94-7			395.2			357.2^*^/225.1			8.56		52		20/27
27	6*α*-fluoro-isoflupredone (6*α*-氟-异氟泼尼龙)				806-29-1			397.2			253.1^*^/263.1			11.42		80		24/29
28	prednisone 21-acetate (泼尼松醋酸酯)				125-10-0			401.2			295.2^*^/147.2			17.89		80		23/39
29	prednisolone-21-acetate (泼尼松龙醋酸酯)				52-21-1			403.3			307.2^*^/367.3			18.20		85		20/15
30	cortisone acetate (可的松醋酸酯)				50-04-4			403.3			163.3^*^/343.3			18.23		140		35/27
31	hydrocortisone acetate (氢化可的松醋酸酯)				50-03-3			405.3			309.2^*^/120.8			18.17		80		25/24
32	beclomethasone (倍氯米松)				4419-39-0			409.2			147.1^*^/279.1			18.15		106		39/41
33	diflorasone (双氟拉松)				2557-49-5			411.2			253.1^*^/235.1			16.17		80		29/37
34	flumethasone (氟米松)				2135-17-3			411.3			253.2^*^/121.0			16.43		80		22/50
35	clobetasol (氯倍他索)				25122-41-2			411.2			373.3^*^/390.9			21.24		90		20/15
36	meprednisone acetate (甲泼尼松醋酸酯)				1106-03-2			415.1			327.2^*^/147.1			21.01		120		22/34
37	desonide (地索奈德)				638-94-8			417.2			323.3^*^/225.1			19.17		80		17/30
38	methylprednisolone acetate (甲基泼尼松龙醋酸酯)				53-36-1			417.2			253.2^*^/399.2			21.49		80		28/28
39	16*α*-hydroxyprednisonlone acetate				86401-80-1			419.2			401.2^*^/323.1			13.21		90		17/18
	(16*α*-羟基泼尼松龙醋酸酯)																	
40	eflone (氟米龙醋酸酯)				3801-06-7			419.3			279.2^*^/321.2			21.72		80		20/19
41	isoflupredone acetate (异氟泼尼松醋酸酯)				338-98-7			421.2			401.3^*^/383.3			17.24		90		13/16
42	fludrocortisone acetate (氟氢可的松醋酸酯)				514-36-3			423.4			239.2^*^/343.2			17.79		170		35/30
43	mometasone (莫美他松)				105102-22-5			427.2			409.3^*^/237.4			22.03		100		17/35
44	budesonide (布地奈德)				51333-22-3			431.2			413.2^*^/147.1			23.73		40		15/33
45	hydrocortisone-17-butyrate (氢化可的松丁酸酯)				13609-67-1			433.3			345.0^*^/120.8			22.96		80		22/23
46	triamcinolone acetonide (曲安奈德)				76-25-5			435.3			415.3^*^/357.2			18.08		100		15/20
47	flunisolide (氟尼缩松)				3385-03-3			435.2			321.2^*^/397.1			18.94		80		18/19
48	betamethasone 21-acetate (倍他米松醋酸酯)				987-24-6			435.3			337.0^*^/309.0			20.77		80		15/15
49	dexamethasone-17-acetate (地塞米松醋酸酯)				1177-87-3			435.3			337.0^*^/309.0			21.26		80		17/15
50	paramethasone acetate (帕拉米松醋酸酯)				1597-82-6			435.2			319.1^*^/171.1			21.47		75		19/40
51	flurandrenolide (氟氢缩松)				1524-88-5			437.3			361.2^*^/285.2			19.38		80		24/29
52	9-fluoro-16*α*,17-(isopropylidenedioxy)corticosterone (氢化曲安奈德)				1524-86-3			437.2			341.1^*^/283.1			19.70		120		30/36
53	deflazacort (地夫可特)				14484-47-0			442.3			123.9^*^/141.9			20.72		80		45/65
54	halometasone (卤美他松)				50629-82-8			445.2			154.8^*^/287.0			18.61		80		45/34
55	prednisolone 21-trimethylacetate (泼尼松龙戊酸酯)				1107-99-9			445.3			427.2^*^/307.2			24.94		90		18/25
56	cortisol 17-valerate (氢化可的松戊酸酯)				57524-89-7			447.3			345.3^*^/121.1			24.50		80		19/39
57	betamethasone 17-propionate (倍他米松丙酸酯)				5534-13-4			449.2			355.2^*^/279.2			22.93		90		16/24
58	fluocinolone acetonide (氟轻松)				67-73-2			453.2			337.1^*^/121.0			18.30		80		21/46
59	flumethasone-17-acetate (双氟美松醋酸酯)				2823-42-9			453.1			253.2^*^/335.3			20.34		110		25/20
60	halcinonide (哈西奈德)				3093-35-4			455.3			377.3^*^/341.2			24.58		170		33/33
61	desonide 21-acetate (21-醋酸地索奈德)				25092-25-5			459.3			441.3^*^/323.1			23.90		100		16/23
62	prednisolone tebutate (泼尼松龙丁醋酸酯)				7681-14-3			459.3			441.2^*^/307.2			25.65		90		16/27
63	prednisolone succinate (泼尼松龙半琥珀酸酯)				2920-86-7			461.2			307.2^*^/147.1			17.37		80		22/37
64	hydrocortisone (醋丙氢可的松)				74050-20-7			461.2			387.2^*^/309.3			23.21		80		15/26
65	fluocortolone pivalate (氟可龙特戊酸酯)				29205-06-9			461.3			443.4^*^/321.2			26.14		110		17/20
66	hydrocortisone hemisuccinate hydrate (氢化可的松琥珀酸酯)				83784-20-7			463.3			327.2^*^/309.3			17.33		145		23/25
67	tixocortol pivalate (替可的松特戊酸酯)				55560-96-8			463.2			343.2^*^/361.2			25.17		80		26/25
68	clobetasol propionate (氯倍他索丙酸酯)				25122-46-7			467.2			373.2^*^/355.2			24.55		80		18/13
69	loteprednol (氯替泼诺)				82034-46-6			467.2			359.1^*^/265.1			24.65		80		26/17
70	methylprednisolone aceponate (甲基泼尼松龙乙丙酸酯)				86401-95-8			473.3			455.2^*^/381.2			24.40		90		16/17
71	methylprednisolone hemisuccinate (甲基泼尼松龙琥珀酸酯)				2921-57-5			475			457.0^*^/339.0			20.67		80		14/17
72	triamcinolone acetonide 21-acetate (曲安奈德醋酸酯)				3870-07-3			477.2			339.2^*^/321.2			23.31		80		22/23
73	dexamethasone valerate (地塞米松戊酸酯)				33755-46-3			477.3			355.2^*^/279.2			24.61		90		16/26
74	betamethasone 17-valerate (倍他米松戊酸酯)				2152-44-5			477.2			355.3^*^/279.3			25.23		80		18/24
75	dexamethasone 21-pivalate (地塞米松特戊酸酯)				1926-94-9			477.3			355.1^*^/337.1			25.69		100		20/20
76	triamcinolone diacetate (曲安西龙双醋酸酯)				67-78-7			479.2			441.2^*^/321.1			15.12		81		14/19
77	clobetasone butyrate (氯倍他松丁酸酯)				25122-57-0			479.3			343.2^*^/279.2			25.36		86		24/24
78	diflucortolone valerate (双氟可龙戊酸酯)				59198-70-8			479.2			375.3^*^/439.4			26.05		80		18/17
79	fluocinonide 22 methyl homologue (醋酸氟轻松22-甲基同系物)				51333-59-6			481.2			441.2^*^/253.1			21.56/21.87		90		21/30
80	halobetasol propionate (卤倍他索丙酸酯)				66852-54-8			485.2			391.2^*^/353.1			24.34		80		17/20
81	prednisolone valerate acetate (泼尼松龙醋酸戊酸酯)				72064-79-0			487.2			367.3^*^/469.3			25.28		120		18/14
82	hydrocortisone cypionate (氢化可的松环戊丙酸酯)				508-99-6			487.6			327.3^*^/309.3			26.74		170		27/29
83	prednicarbate (泼尼卡酯)				73771-04-7			489.2			381.3^*^/115.1			24.77		80		16/25
84	hydrocortisone butyrate propionate (丙丁酸氢化可的松)				72590-77-3			489.4			401.4^*^/327.3			25.48		120		20/30
85	diflorasone diacetate (二氟拉松双醋酸酯)				33564-31-7			495.3			317.1^*^/307.3			23.09		110		20/25
86	fluocinonide (氟轻松醋酸酯)				356-12-7			495.3			455.4^*^/319.3			23.30		120		20/22
87	flumethasone 21-pivalate (氟米松特戊酸酯)				2002-29-1			495.4			477.4^*^/373.3			25.31		110		15/20
88	clocortolone pivalate (氯可托龙特戊酸酯)				34097-16-0			495.3			477.4^*^/457.3			26.43		110		16/20
89	betamethasone benzoate (苯甲酸倍他米松)				22298-29-9			497.3			355.3^*^/337.2			25.55		100		16/20
90	dexamethasone isonicotinate (地塞米松异烟酸酯)				2265-64-7			498.3			478.4^*^/460.4			23.33		110		21/25
91	fluticasone propionate (氟替卡松丙酸酯)				80474-14-2			501.2			313.2^*^/293.2			24.68		80		22/20
92	amcinonide (安西奈德)				51022-69-6			503.4			483.3^*^/399.3			25.28		105		14/17
93	betamethasone 17,21-dipropionate (倍他米松双丙酸酯)				5593-20-4			505.3			319.2^*^/279.1			25.51		71		20/35
94	difluprednate (双氟泼尼酯)				23674-86-4			509.2			303.3^*^/279.3			23.66		110		22/22
95	betamethasone butyrate propionate (倍他米松丁酸丙酸酯)				5534-02-1			519.4			411.2^*^/319.3			26.17		110		18/25
96	alclometasone dipropionate (阿氯米松双丙酸酯)				66734-13-2			521.4			503.3^*^/319.2			24.38		105		13/20
97	mometasone furoate (莫米他松糠酸酯)				83919-23-7			521.3			503.2^*^/355.2			24.63		100		17/24
98	beclomethasone dipropionate (倍氯米松双丙酸酯)				5534-09-8			521.1			503.2^*^/319.2			25.95		80		16/23
99	triamcinolone hexacetonide (己曲安奈德)				5611-51-8			533.3			415.2^*^/397.2			26.97		90		22/23
100	ciclesonide(环索奈德)				126544-47-6			541.4			523.4^*^/305.3			28.49		115		18/28

*Quantitative ion.

## 2 结果与讨论

### 2.1 样品前处理条件的优化

#### 2.1.1 初步提取

取水基质和膏霜乳基质样品考察前处理条件。根据GB/T 24800.2-2009^[6]^及文献[6,14,19,21],考虑到化妆品样品的分散性以及目标化合物的溶解性,对液体(水)类、膏霜乳类化妆品,我们采用饱和氯化钠溶液进行分散后加入乙腈进行提取的方法。饱和氯化钠溶液可脱除水溶性成分,同时有很好的破乳效果,还能有效减少提取体系的乳化效应,减少待测物在水相中的溶解度,从而提高萃取效率。这种饱和氯化钠/乙腈双液相体系还可脱除表面活性剂类成分(分布于双液相界面),而表面活性剂类成分的存在会严重影响目标化合物的离子化效率,脱除此类成分对提高方法的灵敏度至关重要。

#### 2.1.2 加水脱除非极性强的杂质

乙腈提取溶液中仍存在化妆品中蜡基、油脂类等非极性强的成分,会影响色谱柱的使用寿命,干扰目标化合物的检测,需要脱除。有文献使用正己烷脱脂^[5⇓-7]^、QuEChERS^[12,14,21]^、固相萃取^[11,13,17]^等方式净化提取液。正己烷易挥发且对健康和环境有害;QuEChERS成本较高;固相萃取步骤多,效率较低,结果不易平行。按照GB/T 24800.2-2009中的固相萃取条件,以混合标准溶液进行预实验,14种待测物回收率(*n*=2)低于80%,如下:环索奈德(38%)、16*α*-羟基泼尼松龙(39%)、泼尼松龙半琥珀酸酯(43%)、氢化可的松琥珀酸酯(45%)、曲安西龙(50%)、呋曲安奈德(51%)、甲基泼尼松龙琥珀酸酯(59%)、曲安西龙环氧物(64%)、己曲安奈德(70%)、氢化可的松环戊丙酸酯(72%)、美替诺龙醋酸酯(75%)、氢化可的松(75%)、泼尼松龙(76%)、氟泼尼龙(76%)。考虑到向乙腈中加入水,可增加溶液的极性,非极性的蜡基、油脂类成分会析出而被脱除,同时可以消除色谱的溶剂效应。我们向乙腈提取液中加入等体积的水,实验结果显示,乙腈提取溶液中加入水后,有大量沉淀(非极性强的蜡基、油脂类成分)析出。

#### 2.1.3 加入沉淀剂和乙酸

参考GB/T 24800.2-2009^[6]^的样品前处理方法,用亚铁氰化钾溶液和乙酸锌溶液作为絮凝沉淀剂可有效脱除增稠剂等大分子物质,实验证明加入絮凝沉淀剂后的待测液更容易过滤,证实方法有很好的除杂效果。空白加标样品经饱和氯化钠溶液进行分散后,加入乙腈进行提取,再向乙腈提取液中加入等体积的水及亚铁氰化钾溶液和乙酸锌溶液,处理后发现,泼尼松龙半琥珀酸酯、氢化可的松琥珀酸酯、甲基泼尼松龙琥珀酸酯回收率仅为70%左右,考虑到化合物可能因为被沉淀吸附而丢失,尝试通过向提取液中加入酸降低吸附作用提高回收率。实验结果显示,在提取过程中向饱和盐水和乙腈中加入0.2%乙酸后,以上3种目标化合物的回收率均提高至92%以上。为此我们确定了如1.2节所述的样品前处理步骤。

### 2.2 液相色谱-质谱条件优化

#### 2.2.1 色谱柱的选择

本方法包含15组同分异构体,还有一些待测物不是同分异构体,但母离子相同,可能产生相同的子离子,需依靠保留时间来进行定性,为此选择合适的色谱柱是获得良好分离度的关键。文献报道通常使用对苯基化合物有特异选择性的PFP色谱柱及常规C_18_色谱柱对糖皮质激素进行分离。我们考察了实验室常用的有良好分离性能的4种色谱柱对100种待测物的分离效率。在Agilent Poroshell 120 PFP(150 mm×3.0 mm, 2.7 μm)色谱柱上地塞米松戊酸酯和地塞米松特戊酸酯、氟米松特戊酸酯和氟轻松醋酸酯较难分离。在Waters CORTECS T3 C_18_(100 mm×2.1 mm, 2.7 μm)和Agilent Eclipse Plus C_18_ RRHD(100 mm×2.1 mm, 1.8 μm)柱上,异氟泼尼松和氟泼尼龙、地塞米松和帕拉米松较难分离。

Waters ACQUITY UPLC BEH C_18_色谱柱(150 mm×3.0 mm, 1.7 μm)对前述两组同分异构体的分离效果较好,在该色谱柱上,同分异构体泼尼松龙醋酸酯和可的松醋酸酯的保留时间接近,易造成混淆,选择具有特异性的子离子(泼尼松龙醋酸酯307.2/367.3、可的松醋酸酯163.3/343.3),通过离子丰度比可以初步区分,通过MRM-信息依赖采集-增强子离子扫描模式(MRM-IDA-EPI)得到二者的二级质谱图(见图1),可准确分辨泼尼松龙醋酸酯和可的松醋酸酯。

**图 1:**
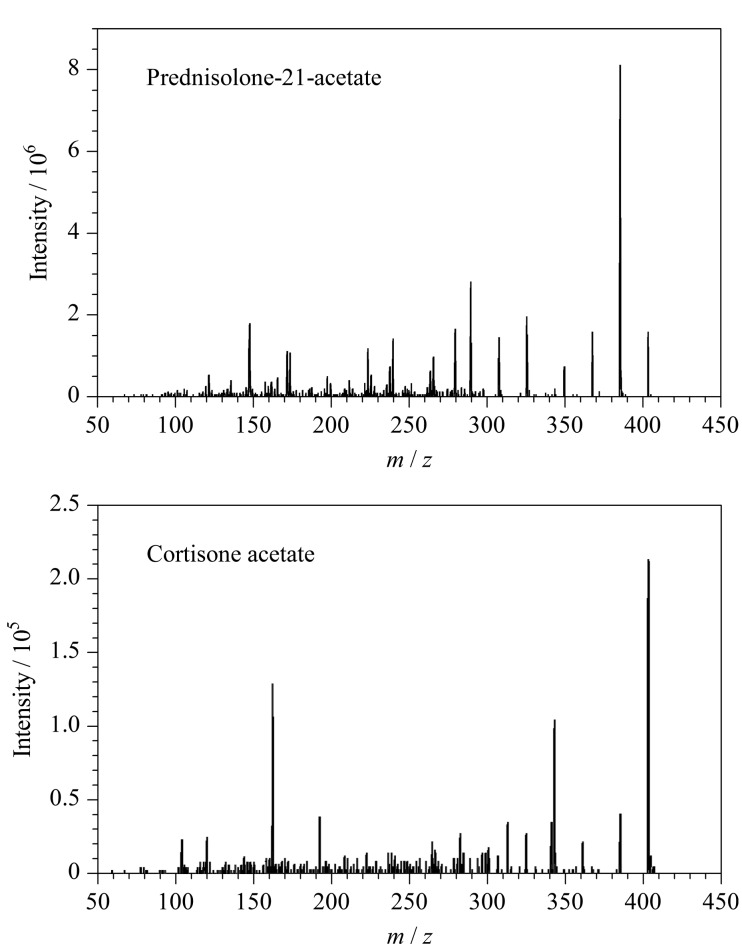
(a)泼尼松龙醋酸酯和(b)可的松醋酸酯的二级质谱图

#### 2.2.2 流动相的选择

我们比较了流动相A相(弱洗脱溶剂)为水,而流动相B相(强洗脱溶剂)分别采用甲醇、乙腈和甲醇-乙腈(1∶1,v/v),梯度洗脱对15组同分异构体目标化合物色谱分离行为的影响。重点考察较难分离的4组同分异构体(氟泼尼龙和异氟泼尼龙、倍他米松和地塞米松和帕拉米松、氟米龙和氟可龙、二氟拉松双醋酸酯和氟轻松醋酸酯),结果表明,甲醇作为流动相B相可获得较好的分离度,最终选择甲醇溶液作为强洗脱溶剂。

在质谱条件探索阶段发现,溶剂中不含氢离子时,部分待测物不能充分离子化而无法检测到母离子,需在流动相中添加酸性改性剂。流动相A和B中同时添加0.1%(v/v)甲酸和0.1%(v/v)、0.2%(v/v)乙酸,实验结果显示,添加0.2%(v/v)乙酸时,响应值最高。在0.2%(v/v)乙酸的水相中分别加入0.5 mmol/L乙酸铵和5 mmol/L乙酸铵,与不加乙酸铵相比,乙酸铵的加入对改善4组同分异构体的分离度没有帮助,且乙酸铵浓度越高,响应值越低。最后确定仅在流动相中添加0.2%(v/v)乙酸以获得更高的检测灵敏度。

#### 2.2.3 质谱条件的优化

在正离子模式下,所有组分均有较高的响应。对待测物进行一级质谱扫描,瑞美松龙和氢化可的松琥珀酸酯的基峰离子为[M-H_2_O+H]^+^,其他98种糖皮质激素均为[M+H]^+^。

对待测物进行二级质谱扫描,综合考虑排除基线干扰、同分异构体的定性区分等因素,选择响应相对较高的两个特征碎片,优化去簇电压(DP)和碰撞能量(CE),确定MRM测定参数。100种糖皮质激素的总离子流色谱图见图2。

**图 2:**
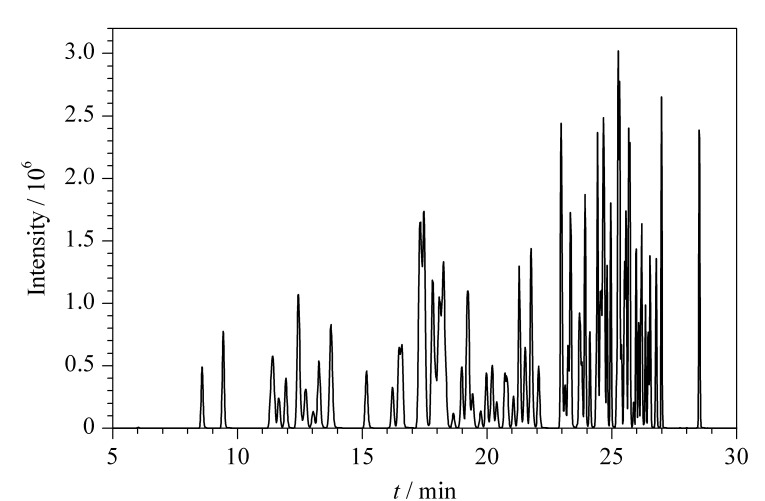
100种糖皮质激素的总离子流色谱图

### 2.3 基质效应

采用提取后添加法配制基质标准溶液,对化妆品中水基质、膏霜乳基质进行基质效应考察,按照公式ME=(1-基质标准曲线的斜率/溶剂标准曲线的斜率)×100%^[12]^ 计算,ME<0表示存在基质增强效应;ME>0表示存在基质抑制效应。绝对值越大则基质效应越明显。结果显示,环索奈德的水基质ME为3.4%,膏霜乳基质ME为30.6%,膏霜乳基质对环索奈德有抑制效应。其余待测物ME为-7.9%~12.4%,99种待测物的基质效应影响不明显,进一步证实了样品前处理方法有效可行。因此,膏霜乳基质中的环索奈德以基质标准曲线定量,膏霜乳基质中的其余99种组分和水基质中的全部100种组分以溶剂标准曲线定量。

### 2.4 方法学考察

#### 2.4.1 线性范围、检出限和定量限

对混合标准工作溶液进行测定,以系列标准溶液质量浓度为横坐标(*x*)、定量离子峰面积为纵坐标(*y*)进行线性回归分析。研究结果表明:各目标化合物在2.5~60 ng/mL范围内具有良好的线性关系,相关系数均大于0.99,详见表2。

**表2:** 100种糖皮质激素的线性方程、相关系数、回收率和精密度

Compound No.	Linear equation	*r*	Water-based (*n*=6)			Cream-based (*n*=6)	
			Recoveries^*^/%	RSDs^*^/%		Recoveries^*^/%	RSDs^*^/%
1	*y*=1.12×10^4^*x*+2.65×10^3^	0.9984	88.6, 111.7, 103.5	5.6, 2.8, 2.5		90.9, 100.6, 94.5	5.5, 6.4, 5.7
2	*y*=5.40×10^4^*x*+2.03×10^3^	0.9994	86.3, 108.0, 94.4	5.7, 3.6, 2.8		98.9, 105.7, 102.2	2.8, 3.0, 1.5
3	*y*=7.26×10^4^*x*+5.57×10^3^	0.9998	86.5, 105.6, 99.2	6.1, 3.1, 3.0		91.4, 98.4, 94.4	3.3, 3.1, 1.5
4	*y*=3.58×10^4^*x*+2.13×10^4^	0.9965	87.1, 109.7, 104.3	4.8, 2.3, 2.0		89.6, 107.5, 105.6	4.7, 2.9, 2.2
5	*y*=9.95×10^3^*x*+6.46×10^2^	0.9977	84.8, 106.7, 94.7	6.3, 4.6, 2.9		99.3, 101.1, 99.5	8.3, 6.7, 2.6
6	*y*=4.29×10^4^*x*+2.14×10^3^	0.9994	97.9, 106.4, 106.2	5.2, 2.9, 1.5		93.0, 98.1, 105.6	3.2, 1.8, 3.4
7	*y*=4.83×10^4^*x*+1.07×10^2^	0.9994	92.3, 105.5, 103.5	5.3, 3.2, 1.8		88.5, 99.6, 98.4	3.8, 3.5, 2.4
8	*y*=8.82×10^4^*x*+8.19×10^3^	0.9989	93.0, 106.4, 102.7	6.8, 2.5, 1.7		86.0, 99.8, 99.9	4.1, 4.1, 2.8
9	*y*=2.34×10^4^*x*+2.37×10^3^	0.9985	91.7, 106.3, 102.6	7.2, 3.4, 2.3		84.5, 101.6, 100.1	3.9, 3.8, 2.9
10	*y*=5.35×10^4^*x*+7.73×10^3^	0.9992	93.9, 111.0, 102.1	5.8, 2.4, 2.6		84.6, 103.2, 93.0	9.2, 3.2, 7.1
11	*y*=3.79×10^4^*x*+1.27×10^4^	0.9981	92.7, 112.8, 105.9	4.3, 1.5, 1.4		87.3, 105.5, 107.4	7.2, 3.9, 1.4
12	*y*=8.62×10^4^*x*+3.55×10^3^	0.9985	94.4, 106.6, 102.5	7.8, 3.6, 2.2		96.0, 104.7, 105.2	6.0, 3.3, 2.1
13	*y*=7.41×10^4^*x*+0.96×10^2^	0.9996	94.9, 94.0, 97.7	5.8, 3.5, 1.9		86.2, 79.5, 91.7	2.9, 6.2, 1.8
14	*y*=1.52×10^4^*x*+1.00×10^4^	0.9970	82.7, 103.8, 102.0	8.5, 4.0, 3.0		91.1, 107.5, 107.4	9.3, 3.9, 3.8
15	*y*=4.95×10^4^*x*+1.68×10^4^	0.9981	87.1, 112.7, 100.0	5.5, 1.6, 1.2		94.6, 110.3, 104.8	2.0, 2.3, 1.7
16	*y*=1.23×10^4^*x*+1.19×10^2^	0.9998	92.9, 108.6, 102.4	6.2, 1.9, 2.3		95.4, 105.9, 103.0	4.4, 2.4, 2.0
17	*y*=1.82×10^4^*x*+7.94×10^2^	0.9998	91.8, 103.9, 101.3	6.1, 2.6, 2.1		98.2, 102.5, 104.5	2.4, 1.9, 1.1
18	*y*=6.87×10^4^*x*-2.60×10^3^	0.9997	100.5, 104.4, 107.3	4.6, 1.9, 1.8		94.3, 98.2, 105.4	3.2, 3.1, 2.3
19	*y*=1.90×10^4^*x*-1.99×10^3^	0.9996	96.9, 105.5, 107.2	5.6, 3.1, 2.4		93.4, 99.0, 106.1	3.6, 1.7, 2.6
20	*y*=1.39×10^4^*x*-1.47×10^3^	0.9999	99.2, 100.1, 106.8	6.4, 3.4, 1.5		93.8, 91.5, 106.2	3.7, 3.1, 2.6
21	*y*=4.39×10^4^*x*+1.26×10^4^	0.9984	84.8, 107.6, 95.0	4.3, 0.9, 2.8		101.3, 103.4, 101.6	3.0, 2.6, 1.9
22	*y*=3.35×10^4^*x*+8.91×10^3^	0.9975	92.1, 107.1, 105.4	6.0, 4.0, 2.5		97.6, 102.6, 105.2	7.1, 3.8, 1.6
23	*y*=7.12×10^4^*x*+1.64×10^4^	0.9978	91.7, 109.1, 103.9	6.1, 3.7, 2.3		93.1, 105.8, 104.9	6.6, 3.9, 2.0
24	*y*=4.38×10^4^*x*+7.62×10^3^	0.9984	98.4, 109.4, 107.0	6.3, 4.2, 2.5		96.8, 102.1, 107.8	5.8, 2.2, 2.4
25	*y*=9.84×10^3^*x*-1.97×10^3^	0.9994	94.0, 107.3, 98.8	4.4, 3.7, 2.2		91.8, 102.6, 101.3	5.1, 2.8, 1.2
26	*y*=4.27×10^4^*x*-4.32×10^3^	0.9995	93.5, 96.6, 99.8	5.2, 1.8, 1.8		90.7, 88.9, 101.5	3.3, 2.8, 1.4
27	*y*=2.92×10^4^*x*+1.44×10^3^	0.9991	96.9, 106.6, 105.6	5.3, 2.0, 1.9		94.5, 100.7, 107.1	4.1, 2.7, 2.2
28	*y*=3.47×10^4^*x*+2.50×10^3^	0.9990	86.9, 108.2, 97.5	4.3, 2.1, 2.1		93.7, 102.1, 100.1	4.7, 2.8, 2.4
29	*y*=2.66×10^4^*x*+1.27×10^4^	0.9971	85.9, 107.6, 100.8	6.0, 3.8, 1.7		93.8, 108.0, 103.4	4.4, 2.7, 1.9
30	*y*=4.07×10^4^*x*+1.63×10^4^	0.9980	83.1, 108.2, 98.2	5.1, 2.2, 2.1		92.3, 104.0, 103.0	4.8, 2.6, 2.1
31	*y*=2.64×10^4^*x*+1.73×10^4^	0.9937	80.5, 110.9, 100.0	7.6, 3.4, 1.8		91.9, 109.2, 104.0	3.6, 2.4, 1.7
32	*y*=7.75×10^3^*x*+4.87×10^3^	0.9928	94.8, 98.0, 103.5	7.2, 4.9, 0.7		88.7, 109.3, 106.6	6.5, 2.6, 1.7
33	*y*=1.28×10^4^*x*-7.76×10^2^	0.9996	92.6, 103.3, 102.0	4.2, 2.4, 2.6		95.0, 104.2, 103.5	2.2, 2.1, 3.4
34	*y*=3.62×10^4^*x*+2.58×10^3^	0.9992	94.2, 107.7, 101.5	6.1, 2.4, 3.5		84.1, 102.6, 92.8	12.0, 2.9, 12.9
35	*y*=5.73×10^4^*x*+9.50×10^3^	0.9983	86.4, 109.6, 99.5	5.0, 2.2, 1.7		96.8, 107.2, 104.0	3.6, 2.3, 2.1
36	*y*=1.88×10^4^*x*-9.66×10^2^	0.9997	85.9, 105.4, 92.1	6.2, 4.0, 2.4		88.2, 105.3, 100.8	5.1, 3.6, 2.2
37	*y*=4.73×10^4^*x*+1.13×10^4^	0.9966	94.4, 111.1, 105.0	6.7, 2.2, 1.7		91.3, 103.9, 104.3	2.7, 1.4, 1.8
38	*y*=1.93×10^4^*x*+6.83×10^2^	0.9989	87.5, 106.0, 98.2	5.5, 1.7, 2.8		104.5, 104.5, 105.5	5.7, 2.6, 1.8
39	*y*=6.78×10^4^*x*-2.80×10^3^	0.9998	92.3, 102.0, 100.9	4.7, 1.9, 2.3		98.1, 98.3, 104.7	3.1, 2.5, 2.1
40	*y*=1.18×10^5^*x*+1.10×10^4^	0.9990	87.4, 109.8, 98.2	6.3, 2.1, 1.6		98.2, 105.1, 102.4	3.5, 1.6, 2.1
41	*y*=5.26×10^4^*x*+1.30×10^4^	0.9988	85.0, 109.6, 97.8	5.2, 2.2, 1.8		99.0, 106.8, 102.6	3.6, 2.1, 2.4
42	*y*=1.02×10^4^*x*+4.03×10^3^	0.9957	84.1, 109.4, 98.0	8.5, 3.9, 1.6		105.8, 101.9, 103.4	7.4, 6.0, 1.8
43	*y*=5.53×10^4^*x*+7.11×10^3^	0.9993	85.9, 107.5, 97.1	5.6, 2.4, 1.5		97.4, 108.7, 103.4	2.2, 2.8, 2.1
44	*y*=8.67×10^4^*x*+1.17×10^3^	0.9995	86.1, 107.0, 93.6	5.8, 2.2, 2.1		96.9, 107.0, 100.5	4.3, 2.9, 2.4
45	*y*=4.99×10^4^*x*+1.40×10^4^	0.9988	86.7, 105.1, 99.0	5.6, 2.8, 2.2		96.5, 106.9, 105.3	4.0, 3.3, 1.8
46	*y*=6.95×10^4^*x*+1.41×10^4^	0.9967	87.9, 102.9, 98.9	5.4, 2.8, 2.0		94.6, 105.0, 107.3	3.4, 3.6, 1.7
47	*y*=4.45×10^4^*x*-2.78×10^3^	0.9993	94.3, 108.6, 102.7	5.9, 2.4, 2.4		94.0, 102.4, 102.5	2.5, 1.2, 1.7
48	*y*=8.99×10^3^*x*+9.66×10^2^	0.9996	83.6, 109.1, 96.8	6.4, 4.0, 1.8		95.5, 104.2, 102.8	5.7, 3.4, 1.4
49	*y*=1.75×10^4^*x*+1.81×10^3^	0.9992	86.8, 105.9, 96.7	6.2, 2.3, 1.2		99.0, 105.8, 103.8	3.2, 5.6, 1.4
50	*y*=2.31×10^4^*x*+3.25×10^3^	0.9996	87.1, 106.5, 98.7	6.6, 1.5, 1.4		98.3, 107.0, 104.3	5.1, 3.0, 1.3
51	*y*=1.68×10^4^*x*-1.52×10^3^	0.9997	97.5, 106.8, 103.1	6.9, 3.7, 2.4		91.1, 102.3, 102.3	2.1, 2.8, 2.0
52	*y*=1.07×10^4^*x*+1.17×10^3^	0.9997	82.1, 104.0, 97.7	8.6, 1.7, 1.5		100.1, 98.4, 100.8	3.5, 4.3, 2.4
53	*y*=1.09×10^4^*x*-2.33×10^2^	0.9985	86.6, 105.3, 93.3	6.0, 2.2, 2.6		102.1, 104.6, 101.9	3.5, 3.4, 2.3
54	*y*=1.13×10^4^*x*+8.04×10^2^	0.9997	88.7, 105.2, 102.6	6.3, 2.6, 1.6		98.0, 99.1, 104.4	4.2, 4.0, 2.5
55	*y*=1.07×10^5^*x*-1.45×10^3^	0.9991	84.7, 107.1, 93.8	6.4, 1.5, 2.8		94.9, 107.8, 94.4	4.8, 2.3, 2.4
56	*y*=6.49×10^4^*x*+4.88×10^3^	0.9993	88.6, 107.6, 98.4	5.8, 3.6, 1.6		100.9, 101.2, 103.6	2.6, 3.3, 1.0
57	*y*=1.56×10^5^*x*+4.83×10^4^	0.9972	87.0, 109.7, 99.0	6.1, 2.2, 2.0		96.6, 108.1, 104.8	3.4, 1.4, 1.5
58	*y*=3.00×10^4^*x*+3.92×10^3^	0.9985	90.2, 108.4, 102.2	5.3, 2.6, 2.1		96.6, 103.3, 104.8	4.8, 4.3, 2.7
59	*y*=1.62×10^4^*x*-3.42×10^2^	0.9999	86.1, 105.6, 95.5	5.7, 2.6, 1.5		93.2, 105.8, 102.6	5.3, 1.8, 2.0
60	*y*=8.93×10^3^*x*+1.77×10^3^	0.9980	84.3, 108.4, 97.3	6.6, 4.8, 1.3		88.1, 108.2, 103.5	7.3, 4.4, 1.5
61	*y*=1.29×10^5^*x*+1.79×10^4^	0.9990	81.0, 104.6, 93.2	5.0, 2.7, 2.0		91.4, 106.8, 96.6	4.5, 1.4, 4.1
62	*y*=1.09×10^5^*x*-1.83×10^3^	0.9996	85.9, 109.2, 94.9	5.9, 3.6, 2.0		99.5, 106.8, 100.6	3.2, 3.0, 1.5
63	*y*=2.12×10^4^*x*-2.68×10^2^	0.9993	99.5, 110.5, 105.4	6.6, 2.3, 1.7		95.9, 108.7, 104.5	7.4, 3.1, 0.9
64	*y*=3.88×10^4^*x*+2.89×10^3^	0.9996	85.0, 106.9, 95.8	5.9, 2.9, 2.5		98.0, 103.8, 99.0	3.2, 3.5, 1.2
65	*y*=3.23×10^4^*x*-0.37×10^2^	0.9993	84.7, 107.8, 95.1	5.0, 2.2, 2.7		91.7, 101.2, 98.8	5.2, 2.3, 1.2
66	*y*=2.73×10^4^*x*+3.01×10^3^	0.9990	97.5, 108.6, 107.2	7.6, 2.3, 1.8		94.7, 106.6, 105.0	2.7, 2.6, 1.8
67	*y*=1.79×10^4^*x*-1.80×10^3^	0.9992	82.6, 107.4, 91.4	8.8, 3.0, 1.7		97.5, 104.5, 101.9	6.1, 3.4, 3.2
68	*y*=4.98×10^4^*x*+1.08×10^4^	0.9992	82.7, 108.8, 97.0	8.6, 2.9, 1.4		97.0, 104.0, 104.1	3.8, 3.6, 2.8
69	*y*=5.12×10^4^*x*+9.36×10^3^	0.9984	85.4, 110.6, 98.1	8.4, 4.0, 3.0		98.0, 111.4, 105.2	7.2, 3.0, 1.4
70	*y*=9.26×10^4^*x*+2.56×10^4^	0.9983	79.7, 106.4, 94.3	7.2, 2.5, 1.2		98.1, 106.2, 102.4	5.8, 3.8, 2.2
71	*y*=2.87×10^4^*x*-3.48×10^3^	0.9998	91.7, 104.4, 99.3	6.2, 3.1, 2.2		100.3, 107.3, 102.7	6.3, 4.5, 1.0
72	*y*=4.68×10^4^*x*+8.92×10^3^	0.9981	86.6, 108.3, 98.5	4.7, 2.6, 1.9		96.1, 107.7, 100.1	2.8, 3.5, 1.5
73	*y*=4.24×10^4^*x*+5.47×10^3^	0.9979	89.1, 111.5, 98.6	6.1, 0.9, 2.5		97.1, 108.5, 104.0	2.7, 2.6, 1.6
74	*y*=8.01×10^4^*x*-3.76×10^2^	0.9995	88.9, 106.7, 96.7	6.0, 1.5, 1.5		98.0, 106.2, 100.3	3.2, 1.6, 1.1
75	*y*=5.82×10^4^*x*+4.28×10^3^	0.9995	80.3, 105.9, 92.9	6.3, 4.9, 1.3		96.0, 107.2, 97.8	4.3, 1.9, 1.8
76	*y*=5.12×10^4^*x*+4.08×10^3^	0.9990	86.8, 106.8, 98.1	5.5, 2.0, 1.2		98.3, 101.9, 102.8	4.3, 2.7, 2.2
77	*y*=3.08×10^4^*x*-3.61×10^2^	0.9987	86.7, 105.8, 94.9	5.7, 2.3, 2.5		104.4, 106.1, 99.5	5.0, 3.9, 2.7
78	*y*=3.49×10^4^*x*-2.10×10^3^	0.9995	84.5, 109.1, 95.2	6.3, 1.3, 2.1		102.0, 105.6, 99.5	6.0, 2.8, 1.6
79	*y*=2.68×10^5^*x*+1.67×10^3^	0.9993	87.6, 107.3, 97.7	6.2, 3.5, 2.4		96.9, 104.3, 101.8	4.2, 2.7, 1.7
80	*y*=2.15×10^4^*x*+2.52×10^3^	0.9995	81.1, 108.7, 91.9	6.4, 4.5, 1.5		96.6, 106.0, 97.7	3.6, 1.9, 2.3
81	*y*=6.34×10^4^*x*+2.53×10^4^	0.9972	79.9, 110.3, 96.8	7.5, 2.0, 2.1		95.8, 110.0, 99.1	5.3, 3.2, 2.1
82	*y*=1.83×10^4^*x*-2.41×10^2^	0.9995	83.1, 107.1, 95.5	7.1, 1.9, 2.7		95.3, 102.2, 93.9	4.4, 5.7, 1.3
83	*y*=8.21×10^4^*x*+2.05×10^3^	0.9989	83.2, 103.8, 92.7	6.0, 5.6, 1.4		93.8, 102.7, 98.8	5.6, 3.7, 1.5
84	*y*=6.38×10^4^*x*-4.05×10^2^	0.9993	86.2, 108.9, 96.4	6.3, 4.8, 3.5		98.7, 106.5, 99.3	4.4, 3.1, 1.7
85	*y*=2.97×10^4^*x*+4.26×10^3^	0.9992	83.6, 107.8, 96.0	6.5, 2.8, 2.2		98.4, 107.6, 102.3	4.6, 2.1, 1.5
86	*y*=1.58×10^4^*x*+4.51×10^3^	0.9980	87.5, 113.8, 99.0	5.8, 1.3, 1.0		92.1, 112.1, 101.0	3.1, 3.4, 3.1
87	*y*=1.45×10^4^*x*+6.08×10^3^	0.9982	80.0, 112.0, 97.9	9.4, 2.7, 2.2		93.7, 107.1, 105.9	5.6, 5.1, 1.7
88	*y*=3.36×10^4^*x*-0.79×10^2^	0.9996	83.9, 107.1, 94.7	7.4, 3.8, 1.9		104.2, 109.5, 99.3	3.8, 3.0, 3.2
89	*y*=7.14×10^4^*x*+2.47×10^2^	0.9988	90.0, 108.8, 96.4	5.4, 2.7, 1.7		99.0, 107.0, 101.9	6.6, 2.5, 0.7
90	*y*=6.94×10^4^*x*+1.85×10^4^	0.9983	83.6, 101.6, 95.6	7.3, 1.7, 2.1		84.9, 94.3, 94.6	5.1, 2.4, 2.4
91	*y*=4.77×10^4^*x*+6.96×10^3^	0.9988	84.9, 109.2, 96.7	7.2, 1.6, 1.8		97.4, 106.8, 103.6	4.4, 3.3, 1.2
92	*y*=6.10×10^4^*x*+2.75×10^4^	0.9955	81.3, 114.4, 98.3	8.4, 2.7, 2.3		97.6, 111.7, 103.4	5.1, 3.2, 1.2
93	*y*=2.81×10^4^*x*+3.21×10^3^	0.9993	81.3, 107.2, 96.7	7.8, 5.4, 1.9		98.1, 104.7, 98.5	2.6, 2.2, 0.9
94	*y*=3.83×10^4^*x*+8.97×10^3^	0.9989	81.1, 100.2, 95.2	5.1, 0.9, 2.2		100.0, 107.9, 102.8	2.5, 2.0, 1.9
95	*y*=7.20×10^4^*x*+1.10×10^4^	0.9994	81.1, 105.3, 97.1	6.3, 2.1, 2.8		97.1, 105.2, 99.6	5.3, 2.0, 1.6
96	*y*=1.95×10^4^*x*+5.24×10^3^	0.9984	79.6, 106.1, 93.7	8.4, 2.4, 2.0		94.3, 107.9, 103.2	7.2, 3.3, 1.2
97	*y*=4.78×10^4^*x*+1.76×10^4^	0.9970	79.7, 110.4, 96.5	8.5, 3.4, 1.7		96.0, 111.6, 102.6	1.5, 0.9, 1.2
98	*y*=8.82×10^4^*x*+9.81×10^3^	0.9989	82.5, 105.4, 96.3	7.8, 2.1, 2.2		97.6, 105.2, 99.6	4.1, 2.3, 2.1
99	*y*=1.15×10^5^*x*+9.19×10^3^	0.9996	82.5, 108.6, 94.7	6.5, 2.9, 1.8		94.6, 105.0, 96.9	3.2, 1.7, 0.9
100	*y*=1.65×10^5^*x*+3.65×10^4^	0.9998	81.6, 105.9, 98.4	9.2, 2.2, 3.6		88.1, 100.0, 80.9	2.5, 1.2, 1.3

Nos. are the same as in Table 1. *y*: peak area; *x*: mass concentration, ng/mL. * Values from left to right represented the results of three spiked levels: 1, 2 and 8 times of LOQs.

100种目标化合物在质量浓度为2.5 ng/mL时定量及定性离子对的信噪比在13~700范围内。虽然不同化合物的质谱响应相差较大,但在标准方法起草过程中通常会综合考虑各种因素,如仪器设备的品牌、型号、仪器状态均会影响化合物的质谱响应,还需要考虑实际应用时化合物的可能浓度以及监管的实际意义,为方便起见一般会在满足质谱响应最低的目标化合物的基础上将检出限、定量限指标统一到一个数值。本文参考《化妆品安全技术规范》^[5]^和GB/T 24800.2-2009^[6]^,最终确定所有目标化合物的方法定量限为0.1 μg/g,方法检出限为0.03 μg/g,定量质量浓度为2.5 ng/mL,检出质量浓度为0.8 ng/mL。

#### 2.4.2 方法回收率及准确度

向不含目标物的水、膏霜乳基质中定量添加目标化合物的标准溶液(3个水平:定量限、2倍定量限和8倍定量限),进行加标回收试验,平行测定6次,结果见表2。水基质中目标物的回收率为79.6%~114.4%, RSD为0.7%~9.4%(*n*=6),膏霜乳基质中目标物的回收率为79.5%~112.1%, RSD为0.7%~12.9%(*n*=6)。

### 2.5 样品筛查

对市售47份化妆品样品(其中膏霜乳25份、爽肤水22份)进行了糖皮质激素的定性和定量分析。

在3份膏霜乳样品中检出5种糖皮质激素,对阳性样品进行两次独立测定,5个目标物两次测定结果的相对偏差均小于10%,含量平均值如下:曲安奈德115.1 μg/g,曲安奈德醋酸酯0.6 μg/g,氯倍他索丙酸酯24.5 μg/g,地塞米松2.7 μg/g,地塞米松醋酸酯210.3 μg/g。按1.4节的色谱分析条件,通过MRM-IDA-EPI扫描模式进行确证,阳性样品溶液以MRM模式锁定符合表1中保留时间及定量离子对的疑似阳性目标物、通过IDA筛选功能触发EPI获得高灵敏度的二级图谱,经SCIEX OS软件的Library Search功能判定,与对照品溶液的EPI质谱图匹配度(Purity)均大于96%。样品中发现的糖皮质激素均是法定标准检验方法涵盖的种类,新型糖皮质激素尚未发现。

## 3 结论

本文采用超高效液相色谱-串联质谱技术,在30 min内实现了化妆品中100种糖皮质激素的同时检测。样品前处理方法简单、有效。方法成本低,效率高,适用于化妆品中100种糖皮质激素的同时定性定量筛查分析,可填补化妆品中糖皮质激素的监管空白,大大提高监管能力,为化妆品的质量监管提供更有力的技术。

## References

[1] Liu H, Zhang J Y, Hu B, et al. Flavour Fragrance Cosmetics, 2020(4): 83

[2] Dong Y L, Qiao Y S, Huang C F, et al. Detergent & Cosmetics, 2020, 43(5): 28

[3] China Food and Drug Administration. Bulletin on the one Batch of Cosmetics Detected Prohibited Raw Materials (No. 54 in 2021). (2021-07-30)[2024-03-03]. https://www.nmpa.gov.cn/xxgk/ggtg/hzhpggtg/hzhpchjgg/hzhpcjgjj/20210730164533187.html https://www.nmpa.gov.cn/xxgk/ggtg/hzhpggtg/hzhpchjgg/hzhpcjgjj/20210730164533187.html

[4] China Food Bulletin on the three Batches of Cosmetics Detected Prohibited Raw Materials (No. 9 in 2024).(2024-02-04)[2024-03-03].https://www.nmpa.gov.cn/xxgk/ggtg/hzhpggtg/hzhpchjgg/hzhpcjgjj/20240204152921101.html https://www.nmpa.gov.cn/xxgk/ggtg/hzhpggtg/hzhpchjgg/hzhpcjgjj/20240204152921101.html

[5] State Food Safety and Technical Standard for Cosmetics (2015 Ed). Beijing: China Standard Press, 2015

[6] GB/T 24800.2-2009

[7] GB/T 40145-2021

[8] Weng D H, Liu J, Jiang Q Q, et al. Flavour Fragrance Cosmetics, 2019(3): 45

[9] Yang P P, Huang W, Li L X, et al. Chinese Journal of Chromatography, 2023, 41(3): 250 10.3724/SP.J.1123.2022.06010 PMC9982709 36861208

[10] Zhao Q R, Liu H, Meng Y P, et al. Chinese Journal of Chromatography, 2023, 41(12): 1084 10.3724/SP.J.1123.2023.04009 PMC10719809 38093538

[11] Ma L B, Sun Y, Zhu G F, et al. China Surfactant Detergent & Cosmetics, 2023, 53(7): 849

[12] Luo H T, Huang X L, Wu H Q, et al. Chinese Journal of Chromatography, 2017, 35(8): 816 10.3724/SP.J.1123.2017.04005 29048815

[13] Liu H, Yang P P, Li L X, et al. Journal of Instrumental Analysis, 2020, 39(9): 1112

[14] Zhao Y, Wang T, Hua L, et al. Journal of Food Safety and Quality, 2019, 10(6): 1559

[15] He J W, Wen J X, Liu Y X, et al. Chinese Journal of Chromatography, 2022, 40(3): 253 10.3724/SP.J.1123.2021.07006 PMC9403998 35243835

[16] Zhang Q Y, Yang H S, Shen M F, et al. Journal of Food Safety and Quality, 2020, 11(15): 5007

[17] Li Y J, Zhao X L, Cen L Y, et al. Flavour Fragrance Cosmetics, 2018(1): 58

[18] Xu Z D, Chen S B, Gao L, et al. Flavour Fragrance Cosmetics, 2019(5): 38

[19] Li H Y, Liu Y, Wang W, et al. Chinese Journal of Analysis Laboratory, 2020, 39(12): 1443

[20] Luo H T, Huang X L, Wu H Q, et al. Chinese Journal of Analytical Chemistry, 2017, 45(9): 1381

[21] Fan X L, Wu W Q, Huang K, et al. Journal of Food Safety and Quality, 2021, 12(1): 86

